# Comparison of Research Knowledge in Postgraduate Trainees of Clinical and Basic Health Sciences of a Tertiary Healthcare Setup: A Qualitative and Quantitative Assessment

**DOI:** 10.7759/cureus.75251

**Published:** 2024-12-06

**Authors:** Akhtar Ali, Farkhunda A Jaleel, Sadia A Waqar, Sehrish Ahmed, Noor Israr, Syed Wajid Shah

**Affiliations:** 1 Pharmacology, Ziauddin Medical College, Ziauddin University, Karachi, PAK; 2 Psychiatry and Behavioral Sciences, Jinnah Postgraduate Medical Center Karachi, Karachi, PAK; 3 General Medicine, Sindh Institute of Physical Medicine and Rehabilitation, Karachi, PAK; 4 Pathology, Ziauddin University, Karachi, PAK; 5 Dental Material, Baqai Dental College, Baqai Medical University, Karachi, PAK; 6 Pharmacy Practice, Ziauddin University, Karachi, PAK

**Keywords:** basic health sciences, clinical sciences, comparison, postgraduate, quantitative and mixed methods research, research knowledge

## Abstract

Background: All recent advances in healthcare, including diagnostics, surveillance, management, and disease prevention, have depended on good-quality research that has brought new information to light. Therefore, in Pakistan, it is important to develop good research skills as, for many years, our physicians have relied on research knowledge from the Western world, which does not necessarily provide solutions to a developing country. Considering the gap in research knowledge among young doctors, the study was planned to compare the research knowledge of postgrad trainees of clinical and basic health sciences (BHS) of private tertiary care hospitals in Karachi.

Methodology: A mixed-method study was conducted in a tertiary care hospital in Karachi. As per the calculated sample size (n=35/group), postgraduate students belonging to BHS and clinical sciences enrolled in research courses were randomly enrolled in the study. Quantitative assessment was carried out by a self-developed questionnaire in which questions regarding the research were asked and qualitative analysis was carried out by interviewing the participants of both groups.

Results: A significant number of participants responded that research was a major part of their coursework, though 20% of participants mentioned that it should be kept as an optional course to participate. When the students were asked the question "Does their supervisor help them with the research?", 21 (60%) participants of BHS responded as "yes", while only 15 (42.9%) participants from the clinical side answered "yes". When it was asked "Does their supervisor have basic knowledge of research", 30 (85.7%) participants from the BHS group responded "yes" (p-value = 0.001).

Conclusion: Both groups had basic knowledge of research and statistical analysis; however, postgraduates from BHS performed better than postgraduates of clinical health science. Postgraduates of clinical health sciences have mentioned that, due to workload and different duty timings, they are unable to participate in research actively. The qualitative assessment highlighted that the students of both sides have been given proper training regarding the research process. Practices of BHS in topic selection and conducting research process seemed to be convincing; however, respondents of clinical science seemed to be unhappy with the practices they followed in their departments.

## Introduction

According to the WHO, research can be defined as the search for new knowledge through scientific methods, investigations, experimentation, and developing new methods for the best use of existing facts and creating new concepts [1,2]. All recent advances in healthcare, including diagnostics, surveillance, management, and prevention of diseases, have depended on good quality research, which has brought new information to light. Hence, it proves that medical research is crucial for improving clinical practice and plays a vital role in updating physicians regarding new developments in the field of medicine [3]. Although the number of medical graduates has increased in Pakistan over recent years and so has the research funding from the Pakistan Medical Research Council (PMRC), it has not been enough to provide significant development and expertise in the field of research. A reason that has been postulated for the shortage of evidence-based research in Pakistan has been the low number of clinician-scientists and the lack of an MD-PhD degree, which has proven useful for better research quality in the United States of America (USA) [4-6]. In 2024, Higher Education Commission of Pakistan (HEC) data showed that Pakistan had 1,351 PhD graduates in the biomedical sciences compared to the USA. In the academic year 2014-2015, 5,282 candidates enrolled in the MD-PhD programs [7,8].

A previous study conducted in Pakistan showed that the knowledge and attitude towards research was insufficient in both private and government trainees, which means a serious lack of research training in health sciences across the country. Two previous studies have shown that multiple factors, such as lack of stipends for conducting research, long working hours, and an inadequate number of research activities, contribute to the lack of knowledge among residents about research work [3,9]. The same study also showed that residents from private medical universities had better knowledge and attitude toward research as compared to government universities possibly because of better undergraduate training in the private sector. It is important to come up with good research ideas and design good research methodologies to achieve the objectives as for many years our physicians have relied on research knowledge from the Western world, which does not necessarily provide solutions to a developing country [9]. For example, one study claims that certain ethnic groups such as South Asians developed diabetes 10 years before the Caucasian population and that recently the incidence of type 2 diabetes mellitus has been increasing rapidly in developing countries [10].

A reason that has been postulated for the scarcity of quality research has been faulty methodology and substandard training on research at the basic level; hence, measures need to be taken to highlight and address these issues that will encourage postgraduate students to conduct more high-quality research [11]. A lack of research interest among clinical residents has been observed because they prioritize clinical training over research training. Conversely, M.Phil. candidates focus on topics related to basic health sciences (BHS), so the study design they mostly attempt is descriptive. However, both the groups attended the same courses of research arranged by the organization while performing the research they were found to be chaotic in identifying the study design, test of significance, sample size, and other research-related parameters. Hence, this study aims to compare the research knowledge of postgrad trainees of clinical and BHS at private tertiary care hospitals in Karachi, Pakistan.

## Materials and methods

Study design and setting

It was a mixed methods study conducted at a tertiary healthcare setup in Karachi from January 2023 to March 2024 after approval from the Ethical Review Committee (Reference Code: 4601221AAPHA).

Sample size and recruiting

The total calculated sample size was n=35, calculated at 50% proportion of the total population of postgraduates studying in clinical and BHS. The sample was recruited by a non-probability consecutive sampling technique.

Inclusion and Exclusion Criteria

Postgraduates enrolled in FCPS, MD, MS, M.Phil., and Masters (associated with medical health sciences) who have been enrolled and completed their research courses or workshops and given consent to participate in the study. The set exclusion criteria were postgraduates who have not completed their research courses and postgraduates of other health-related fields (pharmacy, DPT, nursing).

Data collection procedure for the quantitative analysis

At the end of the session, a self-developed questionnaire was administered to assess the knowledge of postgraduates regarding research. It was a scenario-based questionnaire validated by a group of 10 individuals and reviewed by the associate professor who teaches research at the university. There were 19 multiple choice questions, 13 were regarding knowledge of basic concepts such as study design, and six were regarding basic concepts of statistical analysis that included questions about tests of significance in different scenarios. The total time given to students was 25 minutes, five minutes for filling out the demographic part, and 20 minutes were given to the postgraduates to fill out the questionnaire. The data were collected by investigators on proformas to avoid bias and access to books, notes, or online approaches. The investigator first asked for consent, and after agreement, they provided the questionnaire to study participants.

Data analysis

Data were analyzed using Statistical Product and Service Solutions (SPSS, version 20; IBM SPSS Statistics for Windows, Armonk, NY). Frequency and percentages were calculated for the responses. The chi-square test was used to find the association between groups. p-value less than 0.05 was considered significant.

Data collection procedure for the qualitative assessment

For the qualitative assessment, group-based discussion sessions were organized, invitations were sent to the study participants who participated in the quantitative part, and two online meetings were arranged separately for both groups. To accommodate the timing issues, the meetings were scheduled online at Google Meet. Participants of both groups were asked the same questions, and sessions were monitored by the principal investigator and one member of the research audit committee (RAC). Participants were given five questions based on themes related to research practices. For each question, a 15-minute discussion was allowed, and the principal investigator was responsible for making sure that the discussion followed the theme. When the discussion was diverted to unnecessary arguments, the bell rang, and a new question was asked.

The results of the qualitative part of the study were recorded on individual opinion bases and conclusive remarks by all the participants, and their agreement on the asked theme was assessed and used for inference generation.

## Results

Quantitative assessment

**Figure 1:**
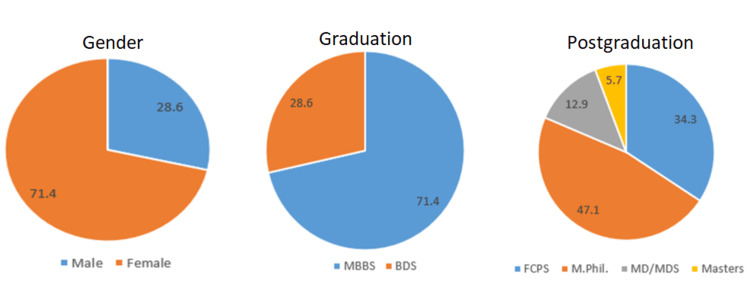
Demographic representation of the study participants in percentage (%).

There were 70 participants in the study belonging to clinical sciences (35, 50%) and BHS (35, 50%). Out of 70, 50 (71.4%) were females, 50 (71.4%) were Bachelor of Medicine, Bachelor of Surgery (MBBS) graduates, and 20 (28.6%) were Bachelor of Dental Surgery (BDS) graduates (36, 51.4%). The mean age of study participants was 28.74 ± 4.1 years. Figure 1 shows the demographic findings of study participants.

When the study participants of both groups were asked about research as part of their postgraduate program, they responded yes (p-value = 0.001; Table 1). Their response was nonsignificant (p-value = 0.082). When they were questioned about whether research writing, data analysis, and publication of an article should be considered an important part of their training, 54 (77.1%) participants marked "yes", while 19 (45.7%) responded as "no". On asking about research writing for money, 16 (45.71%) participants from the clinical group responded "yes" because they think that they do not have much time for research and the same number of participants responded no because this is the major part of their postgraduate program. When they were asked about research training and courses, 29 (82.9%) participants from the BHS group answered "yes". However, 13 (37.1%) participants from the clinical side responded "yes". When asked, "Does their supervisor help them in the research?", 21 (60%) participants of BHS responded "yes", while only 15 (42.9%) participants from the clinical side answered "yes". When it was asked that, "Has their supervisor had basic knowledge of research", 30 (85.7%) participants from the BHS group responded yes (p-value = 0.001). Table 1 shows the responses and chi-square analysis of both groups.

**Table 1:** Responses of students of both groups regarding the importance of research in their postgraduate program. * indicates correct answers.

Question	Group	Yes		No	p-value
Were you taught about research in your postgraduate program?	Clinical	13 (37.1%)		22 (62.9.%)	0.007*
	Basic	29 (82.9%)		6 (17.1%)	
Do you think research should be part of your post-graduate program?	Clinical	26 (74.3%)	9 (25.7%)		0.001*
	Basic	61 (87.1%)	9 (12.9%)		
Should research writing, data analysis, article writing, and publication be considered an important part of a postgraduate program?	Clinical	24 (68.6%)		11 (31.42%)	0.082
	Basic	30 (85.7%)		5 (14.3%)	
According to you is it better to make someone write your research, analyze your data, and make it publishable for money?	Clinical	16 (45.71%)		19 (54.28%)	0.007*
	Basic	4 (11.42%)		31 (88.57%)	
Does your supervisor help in research writing and analysis?	Clinical	15 (42.9%)		20 (57.14%)	0.001*
	Basic	21 (60.%)		14 (40%)	
Does your supervisor understand the basic research concepts of study design, data analysis, and data interpretation?	Clinical	12 (34.3%)		23 (65.7%)	0.001*
	Basic	30 (85.7%)		5 (14.3%)	

Most postgraduates from the BHS group correctly answered the questions regarding basic research study designs. The knowledge of students regarding study design (Questions 2, 3, 6, 7, 11, 12, and 13; Table 2) showed a significant (p-value < 0.05) difference in both the groups and participants from BHS correctly answered the asked scenarios. However, responses regarding clinical trials (Questions 1, 4, 8, 9, and 10) had nonsignificant findings and the majority of participants of both groups marked correct answers. Regarding Question 5, participants of both groups answered wrong, and only two (5.7%) participants from each group marked the correct answer (i.e., selection bias). Table 2 represents the knowledge of postgraduate students regarding basic concepts of research methodology.

**Table 2:** Knowledge of postgraduate students regarding basic concepts of research methodology. * indicates correct answers.

Questions	Options					p-value
1. A study investigating the effect of a new drug for decreasing blood pressure should be a study of which type?		Double-blind placebo-controlled*	Single-blind placebo-controlled	Study without placebo	Triple-blind placebo-controlled	
	Clinical	14 (40.0%)	8 (22.9%)	7 (20.0%)	6 (17.1%)	0.647
	Basic	13 (37.1%)	12 (34.3%)	4 (11.4%)	6 (17.1%)	
2. You are investigating risk factors for a very rare disease. Which type of study you should choose in order to obtain results most effectively and quickly?		Case-control study*	Cross-sectional	Prospective cohort study	Retrospective cohort study	
	Clinical	12 (34.3%)	4 (11.4%)	13 (37.1%)	6 (17.1%)	0.050
	Basic	22 (62.9%)	4 (11.4%)	4 (11.4%)	5 (14.3%)	
3. A community assesses a random sample of its residents by telephone questionnaire. Obesity is strongly associated with diagnosed diabetes. This study design is best described as one of the following		Case-control	Cohort	Cross-sectional*	Experimental	
	Clinical	2 (5.7%)	12 (34.3%)	17 (48.6%)	4 (11.4%)	0.025
	Basic	4 (11.4%)	2 (5.7%)	25 (71.4%)	4 (11.4%)	
4. Treatment A was found to have a significant effect with p-value = 0.05 and the treatment B effect was found significant with p value = 0.002. Based on the p values what is the effect of treatment A and treatment B as compared to each other?		Both treatments have an equal effect	It is impossible to compare the size of the effects	Treatment A has a greater effect	Treatment B has a greater effect*	
	Clinical	7 (20.0%)	4 (11.4%)	13 (37.1%)	11 (31.4%)	0.163
	Basic	2 (5.7%)	8 (22.9%)	10 (28.6%)	15 (42.9%)	
5. When conducting a retrospective cohort study which type of bias is the researcher most likely to experience?		Confirmation	Publishing	Recall	Selection*	
	Clinical	7 (20.0%)	19 (54.3%)	7 (20.0%)	2 (5.7%)	0.001
	Basic	3 (8.6%)	1 (2.9%)	29 (82.9%)	2 (5.7%)	
6. A group of researchers are conducting a study on the effect of raised BMI versus normal BMI on the development of heart disease and will follow the study participants over the course of 10 years. Which type of study is this?		Case-control	Cross-sectional	Prospective cohort*	Retrospective cohort	
	Clinical	13 (37.1%)	6 (17.1%)	10 (28.6%)	6 (17.1%)	0.001
	Basic	6 (17.1%)	1 (2.9%)	28 (80.0%)	0	
7. Which type of study can be used for descriptive epidemiology but does not provide any information on risk factors or disease causality?		Case-control	Cross-sectional*	Prospective cohort	Retrospective cohort	
	Clinical	13 (37.1%)	9 (25.7%)	11 (31.4%)	2 (5.7%)	0.001
	Basic	1 (2.9%)	30 (85.7%)	2 (5.7%)	2 (5.7%)	
8. A medical university is conducting research on the intake of vitamin D in hypertensive patients. They identified a group of people with hypertension and a control group consisting of people without hypertension. Which type of study design can be used for above mentioned protocol?		Case-control*	Cohort study	Cross-sectional	Double blind placebo	
	Clinical	19 (54.3%)	2 (5.7%)	7 (20.0%)	7 (20.0%)	0.557
	Basic	20 (57.1%)	3 (8.6%)	9 (25.7%)	3 (8.6%)	
9. Existing information about a topic can be termed as?		Complex hypothesis	Empirical hypothesis	Null hypothesis*	Simple hypothesis	
	Clinical	4 (11.4%)	13 (37.1%)	14 (40.0%)	4 (11.4%)	0.131
	Basic	0	11 (31.4%)	16 (45.7%)	8 (22.9%)	
10. Sample size of a research based on disease prevalence is calculated by which of the following software/tool?		Open epi.*	PubMed	Sealed envelope	SPSS	
	Clinical	15 (42.9%)	4 (11.4%)	4 (11.4%)	12 (34.3%)	0.251
	Basic	22 (62.9%)	2 (5.7%)	5 (14.3%)	6 (17.1%)	
11. Which one of the following factors should not be considered while selecting a topic?		Acceptability	Controversy*	Cost effectiveness	Feasibility	
	Clinical	1 (2.9%)	16 (45.7%)	5 (14.3%)	13 (37.1%)	0.002
	Basic	4 (11.4%)	27 (77.1%)	0	4 (11.4%)	
12. The results of a study were not significant and all the results were rejecting alternative hypothesis. The researcher must?		Analyze the results again	Change the hypothesis	Not present the results	Present the results and conclude the findings*	
	Clinical	11 (31.4%)	0	9 (25.7%)	15 (42.9%)	0.001
	Basic	4 (11.4%)	4 (11.4%)	0	27 (77.1%)	
13. When writing an article, the rationale and objectives of study should be mentioned in which part of the article?		1st Paragraph	2nd Paragraph	3rd Paragraph*	Methodology	
	Clinical	16 (45.7%)	12 (34.3%)	7 (20.0%)	0	0.023
	Basic	10 (28.6%)	7 (20.0%)	13 (37.1%)	5 (14.3%)	

To assess the knowledge regarding statistical analysis, six scenario-based questions were asked (Table 3). Postgraduates of BHS correctly answered Questions 2, 4, and 5 (questions regarding statistics) with a p-value < 0.05. Questions 1 and 6 were answered wrongly by participants of both the groups, though the majority of participants of the clinical sciences group marked both these questions correctly. Question 3 was answered wrong by the majority of participants of both groups.

**Table 3:** Knowledge of postgraduate students regarding basic concepts of statistical analysis * indcicates correct answers.

Question	Options					p-value
1. Which test of significance should be used for comparison of prevalence of disease A in men and women?		ANOVA	Chi square*	Shapiro-Wilk	Student T-test	
	Clinical	5 (14.3%)	14 (40.0%)	5 (14.3%)	11 (31.4%)	0.245
	Basic	6 (17.1%)	9 (25.7%)	2 (5.7%)	18 (51.4%)	
2. Which test should be used for comparison of blood pressure values between subjects belonging to three levels of smoking?		ANOVA*	Chi square	Shapiro-Wilk	Student T-test	
	Clinical	10 (28.6%)	11 (31.4%)	1 (2.9%)	13 (37.1%)	0.003
	Basic	23 (65.7%)	8 (22.9%)	2 (5.7%)	2 (5.7%)	
3. A researcher compares satisfaction levels from treatment received in emergency department (measured in ascending categories from 1 to 4) between two study groups. Which test should be used?		ANOVA	Chi square*	Independent samples t-test	Paired samples T-test	
	Clinical	16 (45.7%)	3 (8.6%)	3 (8.6%)	13 (37.1%)	0.003
	Basic	7 (20.0%)	8 (22.9%)	13 (37.1%)	7 (20.0%)	
4. An educational intervention was carried out in a group of students via proforma for assessing the difference in knowledge, that was given to them before and after the intervention. Which of the following analysis methods will highlight the pre and post-educational differences in students?		ANOVA	Chi square	Paired T-test*	Shapiro-Wilk	
	Clinical	4 (11.4%)	17 (48.6%)	11 (31.4%)	3 (8.6%)	0.001
	Basic	1 (2.9%)	0	31 (88.6%)	3 (8.6%)	
5. Before performing statistical analysis normal distribution of data is obtained to categorize the data as parametric or non-parametric. Which of the following tests identifies the normal distribution of data?		ANOVA	Chi square	Shapiro-Wilk*	Student T-test	
	Clinical	3 (8.6%)	17 (48.6%)	9 (25.7%)	6 (17.1%)	0.006
	Basic	3 (8.6%)	6 (17.1%)	23 (65.7%)	3 (8.6%)	
6. Australian researchers found that excessive use of sun-protective cream is related to the development of skin cancer. This relationship could be partially explained by the presence of a confounder. To assess the direct effect of the cream on the development of skin cancer, the researchers should perform?		Causal analysis	Descriptive analysis	Multivariable analysis*	Predictive analysis	
	Clinical	11 (31.4%)	7 (20.0%)	17 (48.6%)	0	0.186
	Basic	16 (45.7%)	3 (8.6%)	14 (40.0%)	2 (5.7%)	

Qualitative assessment

From BHS, seven participants responded to the invitation and joined the meeting, and from the clinical sciences group, five participants joined the link. They were asked five questions and their responses were recorded.

Were you given proper education about the process of research?: The majority of participants from the BHS group responded "yes". They mentioned that, during their coursework, they studied research methodology, basic biostatics, and epidemiology as compulsory courses. Furthermore, they discussed that, during the research phase of their degree program, they were assigned a day as their research day. On their research day, they all had an exemption from their academic tasks. Through their discussion, they seemed satisfied with their understanding of the research process.

The participants of the clinical group responded "yes" to the question; however, they mentioned that, due to workload and shift-changing issues, it was difficult for them to attend the research courses on a regular basis. Furthermore, their responses were, as we believe, that attending a session regularly could have improved our understanding of the research process. We know that, in the future, it will be difficult for us to understand and define research for our students.

What are the common practices followed by you or your seniors in selecting and working on the topic?: The respondents of BHS responded that it is mandatory to participate and attend the weekly journal club session. In that session, the BHS trainees present research articles in their subjective domain and get ideas about topic selection, which is further facilitated by the departmental colleagues. Contrary to this, participants in clinical sciences reported that their supervisor and seniors suggested the research topics, which were subsequently approved by the head of the department. They added that, as the topics are being suggested to them by seniors, therefore, concept development regarding that subject area and carrying out further research processes become difficult for them.

Did your supervisors help in research idea development, synopsis writing, data collection procedures, and dissertation/thesis writing?: The BHS respondents said "yes" to the asked question; however, they mentioned that their supervisors are not well trained for data analysis and results interpretations. Hence, they always seek help from statisticians and for some techniques they require assistance from other faculty members who are experts in that field. The clinical health sciences respondents replied "yes" for idea development; as in the last question, they mentioned that idea is given to them by their seniors or supervisors. However, they added that they perform further research processes on their own they pay a high amount to satiations for data analysis and interpretation. They added that, due to busy schedules at clinics, supervisors do not facilitate them as per need; hence, they ask for help from their senior colleagues or friends.

What difficulties have you experienced during the research process?: The respondents of BHS reported that it became difficult for them to match the timeline as, after the selection of a topic as per their university policy, they need approval from the research guidance and evaluation committee, ethics review committee, and board of advanced studies and research. According to them, it takes three to five months on average to get through this process. The candidates for clinical sciences also discussed the same issue. Furthermore, the participants of both sides highlighted the issue of article publication. They mentioned that, in national journals, it takes at least a year to publish an article, and, for international journals, their unaffordable fee becomes another hindrance in their research process. They highlighted that, despite they are being taught about biostatics as that is not their field, they feel difficulty in performing data analysis and interpretation.

In your opinion how can the research process improve?: The BHS respondents mentioned that the university can facilitate them in improving the research process by increasing the subject expert faculty, decreasing the wastage of time in approvals from different committees, and providing funds from the university for research experiments and publication. Contrary to this, the clinical sciences respondents mentioned that the university should allot them a day in week for research. Additionally, they mentioned that their supervisors should specify one day per week only to facilitate their candidates in the research process.

## Discussion

The research students often face difficulties in their research process that delay their studies and extend their completion timeline. These problems arise in research designing, data collection, experimentation, and writing the dissertation. Research knowledge has a pivotal role in the training of postgraduate students in both BHS and clinical practice. However, the same approach in the postgraduate research course of BHS and clinical programs creates variance in their outcome. BHS and clinical postgraduates have different tasks to perform; therefore, their research course should be molded accordingly that facilitate their learning output and not the other way around. The research course comprises of research methodology section along with a study design section, followed by basic biostats teachings. The method of delivery is mostly comprised of lectures and quizzes. Assessment takes place in the form of best-choice questions.

According to our study, clinicians are reluctant to go through the research courses. These results are in line with previous studies demonstrating that students are more active and productive in their research when their supervisors support them in more practical ways. According to Ismail et al. [12], the supervisor’s role is of prime importance in the student’s research. In his study, he found students complaining about less time given by the supervisors in research writing and designing that hampers their research output [12]. The reason for inadequate time could be the busy schedules of supervisors in hospitals. On the other hand, Obuku et al. demonstrated that it is the students who if self-motivated can strongly support their research proposals [13].

Literature revealed that not every student can master the art of statistics. They find it difficult to understand and apply [14]. Our study is novel because we compared the knowledge between the BHS students and clinician students. Therefore, no previous data are available in this regard. We found that the BHS students are more knowledgeable than clinicians when it comes to research statistics skills. This could be because of the more productive time that they can spend on learning and implementing knowledge as compared to the clinicians who are more involved in patient handling. It can also be deduced that postgraduate clinical students face difficulties with their research protocol because of a lack of participation in selecting the research topic. Embarking on the research work without prior preparation may lead to confusion and anxiety [15].

The knowledge of students regarding statistical analysis is of utmost importance. Proper and accurate application of statistical tests needs skills and experience in the research field [16]. Literature has revealed that there is a lack of knowledge in this regard, not only in students but also in supervisors [17]. A lack of knowledge of statistics may lead to erroneous conclusions of the study that may cause false additions to the literature [18,19]. Proper guidance from a supervisor who has sound knowledge of data analysis may help students in such a curious time. If students lack this guidance and knowledge, they go for outsourcing their data and hire special statisticians for this purpose [20]. However, this lack of knowledge can create a reluctance in the postgraduate clinical students for further research [21]. Furthermore, we evaluated the qualitative questions given to both groups. Most of the participants requested to be exempted from their other tasks on the research day so that they could fully focus on the agenda. Multiple respondents from the groups emphasized that timelines to finish the research can be matched if the time between the approvals from different research committees is minimized [22].

Some comparisons can be drawn. Firstly, BHS students were found to be sounder in the knowledge of research as compared to the clinical science group; however, with regard to the statistical course, both groups showed better knowledge. This disparity can be addressed by incorporating evidence-based research curricula for both the clinicians and the BHS students without contributing further to their stress and exhaustion. The novelty of our research is that we made a comparison between these groups. Therefore, data were scarce for reference in this regard. This study will add new data to the literature.

Possible limitations of our study may include the following: (1) the sample size (35 per group) is relatively small and may not adequately represent the diverse experiences of postgraduate students in the country; (2) the use of non-probability consecutive sampling may introduce bias, as the sample might not be representative of the entire postgraduate trainee population; (3) the self-developed questionnaire, though validated, may not comprehensively capture all aspects of research knowledge and skills; (4) and the findings are specific to one institution in Karachi and do not account for variability across other medical institutions in Pakistan.

## Conclusions

Both groups had basic knowledge of research and statistical analysis; however, postgraduates of BHS performed better than postgraduates of clinical health sciences. Postgraduates of clinical health sciences have mentioned that, due to workload and different duty timings, they are unable to participate in research actively. The qualitative assessment highlighted that the students of both sides have been given proper training regarding the research process. Practices of BHS in topic selection and conducting research process seemed to be convincing; however, respondents of clinical science seemed to be unhappy with the research practices they followed in their departments. The BHS students showed confidence in their supervisors, which was contrary to the students of clinical sciences; however, reservations about the knowledge of basic biostatics among the supervisors were reported by both groups. Both the groups reported difficulty in matching the timeline due to wastage of time due to approvals from different committees, article processing and publication, and performing basic biostatics. Both groups mentioned that, by providing enough funds and allocation of research days to candidates and supervisors, the research process can be improved.
